# Production of cold-active pectinases by three novel *Cladosporium* species isolated from Egypt and application of the most active enzyme

**DOI:** 10.1038/s41598-022-19807-z

**Published:** 2022-09-16

**Authors:** Ahmad Mohamed Moharram, Abdel-Naser Ahmed Zohri, Abd El-Latif Hesham, Hossam E. F. Abdel-Raheam, Mohamed Al-Ameen Maher, Osama Abdel-Hafeez Al-Bedak

**Affiliations:** 1 Department of Botany and Microbiology, Faculty of Science, Assiut University, Assiut, 71511 Egypt; 2 Assiut University Mycological Centre, Assiut University, Assiut, 71511 Egypt; 3 Department of Genetics, Faculty of Agriculture, Beni-Suef University, Beni-Suef, Egypt; 4 Department of Food Sciences, Faculty of Agriculture, Beni-Suef University, Beni-Suef, Egypt

**Keywords:** Biotechnology, Microbiology

## Abstract

*Cladosporium parasphaerospermum*, *Cladosporium chlamydosporigenum*, and *Cladosporium compactisporum* have all been discovered and characterized as new *Cladosporium* species. The three new species seemed to generate cold-active pectinases with high activity at pH 6.0 and 10 °C, pH 6.0 and 15 °C, and pH 5.0 and 15 °C, respectively, with the most active being *C. parasphaerospermum* pectinase. In submerged fermentation (SmF), *C. parasphaerospermum* produced the most cold-active pectinase with the highest activity and specific activity (28.84 U/mL and 3797 U/mg) after 8 days. *C. parasphaerospermum* cold-active pectinase was isolated using DEAE-Cellulose anion exchange resin and a Sephadex G 100 gel filtration column. The enzyme was purified 214.4-fold and 406.4-fold greater than the fermentation medium using DEAE-cellulose and Sephadex G 100, respectively. At pH 7.0 and 10 °C, pure pectinase had the highest activity (6684 U/mg), with K_m_ and V_max_ determined to be 26.625 mg/mL and 312.5 U/min, respectively. At 5 mM/mL, EDTA, MgCl_2_, and SDS inhibited the activity of pure pectinase by 99.21, 96.03, and 94.45%, respectively. The addition of 10 U/mL pure pectinase enhanced the yield of apple, orange, apricot, and peach juice by 17, 20, 13, and 24%, respectively, and improved the clarity and colour of orange juice by 194 and 339%, respectively. We can now add cold-active pectinase production to the long list of *Cladosporium* species that have been identified. We also report three new species that can be used in biotechnological solutions as active microbial pectinase producers. Although further research is needed, these distinct species might be used to decompose difficult and resistant pectinacious wastes as well as clear fruit juices.

## Introduction

*Cladosporium* is one of the largest and most complex genera of hyphomycetes, which currently includes more than 728 names. Until recently, all types of unrelated dematiaceous hyphomycetes characterized by amero-to-phragmosporous conidia formed in acropetal chains had been referred to *Cladosporium*^1^. Species of *Cladosporium* are well adapted to spread easily in large numbers over long distances, therefore they are cosmopolitan and widely present in all various types of plants and other debris, mostly isolated from air, soil, seeds, grains, food, paint, textiles and other organic matter^1–9^. Several species of this genus are plant pathogenic causing leaf spots and other lesions^10^, or they occur as hyperparasites on some fungi^11^.

*Cladosporium* species are also known to be common endophytes^12–14^ as well as phylloplane fungi^15–18^. Some *Cladosporium* species including *C*. *cladosporioides*, *C*. *chlorocephalum* and *C*. *uredinicola* were recorded as entomopathogens of Aphids and whiteflies^19,20^. The Genus *Cladosporium* is considered to be a rich source of diverse and bioactive natural compounds^21^. Some species were reported to produce anticancer compounds such as L-asparaginase^22^, paclitaxel^23^ and useful enzymes including cellulases^24^ and pectinases^25–27^.

Enzymes are nowadays in great demand in a variety of industrial applications, including food, detergent, paper, textiles, and organic chemical synthesis, due to their high efficiency and environmental friendliness^28–30^. Furthermore, these enzymes are part of a well-established worldwide industry that is expected to grow to US$6.3 billion by few years^28–31^. The current tendency is to utilize cold-active enzymes to lower the temperature of industrial processes, allowing for energy savings and reduced carbon footprint, as well as the productive capacity that operate better at ambient or lower temperatures^32–34^. Because they are (i) cost-effective, (ii) energy saving, (iii) capable of catalyzing processes without additional heat aid, and (iv) selectively inactivated by mild heat input^35^. In biotechnology, cold-active enzymes are used to prevent a range of unwanted reactions and restrict the loss of volatile components^36–39^. As a result of these inevitable uses, the function of cold-active enzymes is expected to increase dramatically in the next years^39,40^.

Pectin is one of the most numerous and complicated polymers that make up the plant cell wall. It is a group of polysaccharides that contain at least seven structural components, the most well-known of which are homogalacturonan, xylogalacturonan, rhamnogalacturonan I, and rhamnogalacturonan II^41,42^. Pectin is made up of a main chain of galacturonic acid residues bound by (14) links (homogalacturonan), or a mixture of galacturonic acid and rhamnose (rhamnogalacturonans) or galacturonic acid and xylose (rhamnogalacturonans) (xylogalacturonan). Various molecules, such as methyl, ethyl, and various sugar moieties (arabinose, rhamnose, galactose, and others), can then be replaced for the main chain^43^. Pectin biodegradation necessitates the coordinated activity of multiple enzymes known collectively as pectinases, due to its complicated structure. Pectin methyl esterases, pectin acetyl esterases, polygalacturonases, polymethylgalacturonases, polygalacturonate lyases, polymethylgalacturonate lyases, rhamnogalacturonase, arabinases, and xylogalacturonases are all examples of pectinases^42^.

Around 10% of the enzyme market is made up of pectinolytic enzymes, which are used in the juice, food, paper, and textile industries^44–46^. Low temperatures (15 °C) are employed in the juice industry to minimize cloudiness and bitterness in fruit juices in order to prevent the spread of harmful bacteria, preserve labile and volatile flavor components, and save energy^34,45^. Researchers have been looking for pectinases that can act at low temperatures but also at low pH, because the pH of fruit juices and grape must be between 2.5 and 3.5^42^. Pectinases are presently derived from mesophilic filamentous fungi, mostly Aspergillus species, however they work poorly below 35 °C^47^. Cold-active enzymes, on the other hand, have higher enzymatic activity than mesophilic enzymes at lower temperatures^48^. As a result, the current research sought to develop cold-active pectinases from three newly-discovered *Cladosporium* species from Egypt, as well as purify, characterize, and use the most active pectinase in fruit juice production.

## Results

### Molecular studies

#### *Cladosporium parasphaerospermum* AUMC 10865

**ITS:** Based on a megablast search of NCBIs GenBank nucleotide database, the closest hits using ITS sequence are *Cladosporium cladosporioides*, *Cladosporium parahalotolerans* and *Cladosporium halotolerans* [(GenBank KJ767065, MK262909 and MK258720; identities = 554/556 (99.64%); Gaps = 1/556 (0%)]. **ACT:** the closest hits using ACT sequence are *Cladosporium halotolerans* and *Cladosporium omanense* [(GenBank MF084398 and MH716046; identities 127/132 (96.21%); Gaps = 1/132 (0%)]. **LSU:** the closest matches using LSU are *Digitaria exilis* [(GenBank LR792838; identities = 1142/1152 (99.13%); Gaps = 10/1152 (0%)] and *Cladosporium delicatulum* [(GenBank JQ732984 and JQ732983; identities = 1140/1152 (98.96%); Gaps = 10/1152 (0%)].

#### *Cladosporium chlamydosporigenum* AUMC 11340

**ITS:** Based on a megablast search of NCBIs GenBank nucleotide database, the closest hits using ITS sequence are *Cladosporium subcinereum* [(GenBank OK510262; identities = 551/554 (99.46%); Gaps = 2/554 (0%)] and *Cladosporium floccosum* [(GenBank MK460809; identities = 549/553 (99.28%); Gaps = 3/553 (0%)]. **ACT:** the closest hits using ACT sequence are *Davidiella tassiana* [(GenBank EU730605; identities = 229/230 (99.57%); Gaps = 0/230 (0%)] and *Cladosporium herbarum* [(GenBank EF679510 and EF679511; identities = 228/230 (99.13%) and 227/230 (98.70%); 0/230 (0%)]. While compared to the type materials, the closest hits are *Cladosporium macrocarpum* [(GenBank EF679529; identities = 226/230 (98.26%); Gaps = 0/230 (0%)], *Cladosporium herbarum* [(GenBank EF679516; identities = 225/230 (97.83%); Gaps = 0/230 (0%)] and *Cladosporium versiforme* [(GenBank KT600613; identities = 216/227 (95.15%); Gaps = 3/227 (1%)]. **LSU:** the closest hits using LSU sequences are *Cladosporium herbarum* and *Cladosporium allicinum* [(GenBank MH047193 and GU214408; identities = 1184/1199 (98.75%); Gaps = 13/1199 (1%)].

#### *Cladosporium compactisporum* AUMC 11366

**ITS:** Based on a megablast search of NCBIs GenBank nucleotide database, the closest hits using ITS sequence are *Cladosporium cladosporioides* [(GenBank ON045558 and MT367253; identities = 547/552 (99.09%) and 548/554 (98.92%); Gaps = 0/552 (0%) and 1/554 (0%)]. **ACT:** the closest hits using ACT sequence are *Cladosporium cladosporioides* [(GenBank KY886457; identities = 232/232 (100%); Gaps = 0/232 (0%)], *Cladosporium proteacearum* (ex-type) [(GenBank MZ344213; identities = 223/229 (97.38%); Gaps = 0/229 (0%)], *Cladosporium devikae* (ex-type) [(GenBank MZ344212; identities = 212/216 (98.15%); Gaps = 1/216 (0%)] and *Cladosporium cladosporioides* (ex-type) [(GenBank HM148490; identities = 220/230 (95.65%); Gaps = 0/230 (0%)]. **LSU:** the closest hits using LSU sequence are *Cladosporium delicatulum* and *Cladosporium uredinicola* [(GenBank JQ732985 and EU019264; identities = 1206/1221 (98.77%); Gaps = 12/1221 (0%)], *Toxicocladosporium irritans* (ex-type) [(GenBank EU040243; identities = 1184/1221 (96.97%); Gaps = 12/1221 (0%)], and *Rachicladosporium inconspicuum* (type) [(NG_059443; identities = 1159/1224 (94.69%); Gaps = 16/1224 (1%)].

#### Phylogenetic analyses

**Figure 1:**
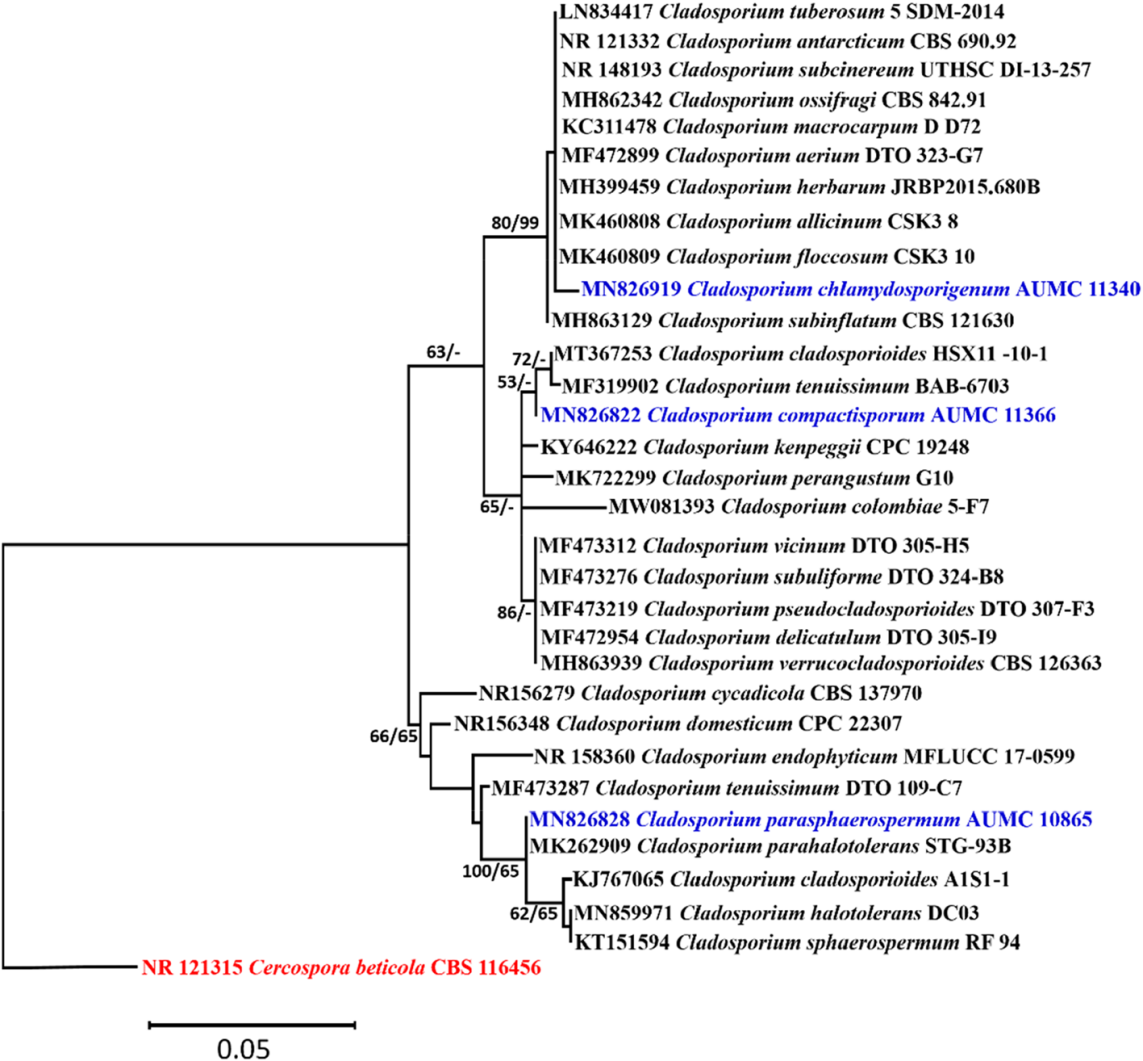
Maximum likelihood phylogenetic tree generated from ML/MP combination analysis based on alignment of ITS sequences of *C. parasphaerospermum* AUMC 10865, *C. chlamydosporigenum* AUMC 11340 and *C. compactisporum* AUMC 11366 with the most similar sequences belonging to *Cladosporium* in GenBank database. Sequences of species in this study are in blue color. Bootstrap support values (1000 replications) for ML/MP combination equal to or greater than 50% are indicated at the respective nodes. The tree was rooted to sequence of *Cercospora beticola* CBS 116456 as outgroup (in red color).

**Figure 2:**
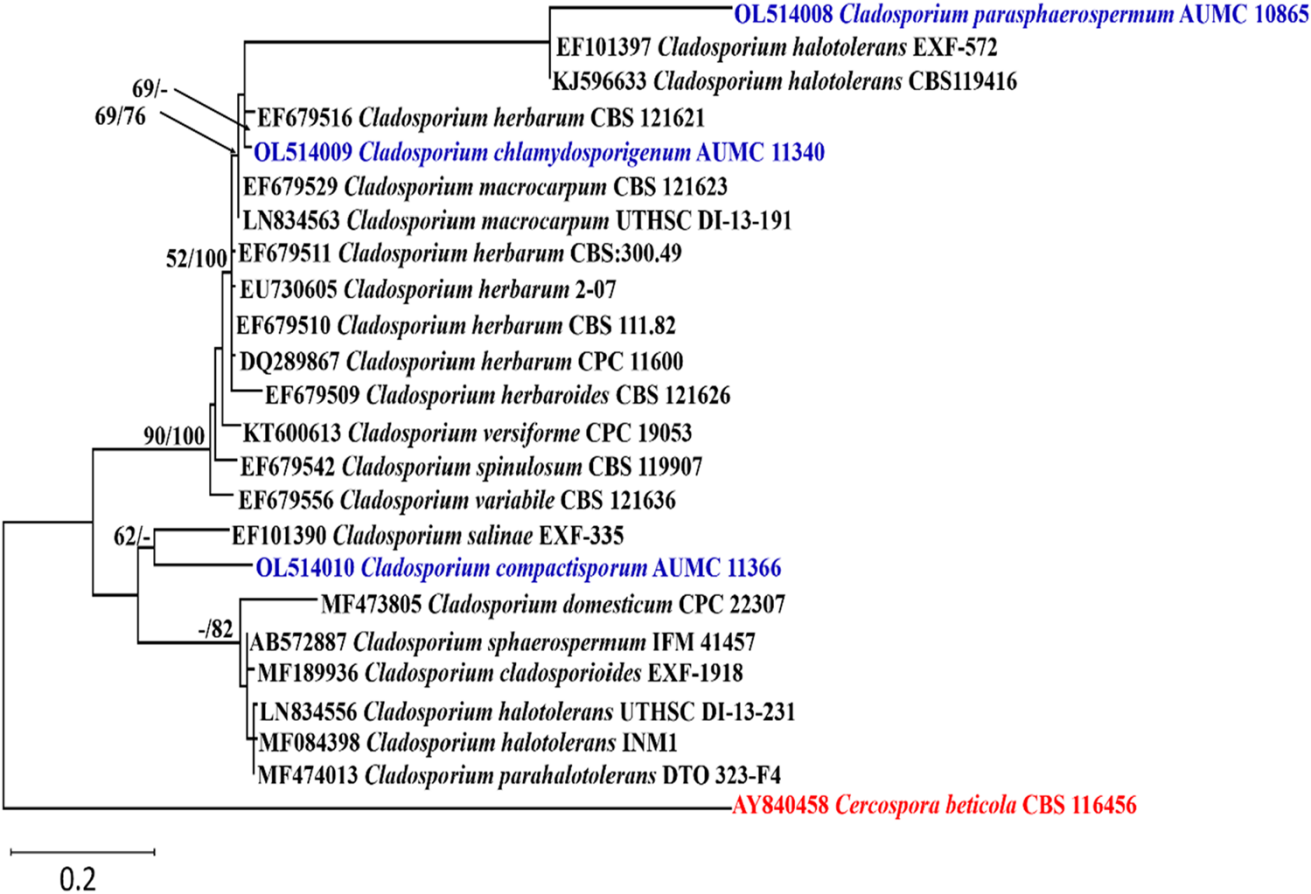
Maximum likelihood phylogenetic tree generated from ML/MP combination analysis based on alignment of ACT sequences of *C. parasphaerospermum* AUMC 10865, *C. chlamydosporigenum* AUMC 11340 and *C. compactisporum* AUMC 11366 with the most similar sequences belonging to *Cladosporium* in GenBank database. Sequences of species in this study are in blue color. Bootstrap support values (1000 replications) for ML/MP combination equal to or greater than 50% are indicated at the respective nodes. The tree was rooted to sequence of *Cercospora beticola* CBS 116456 as outgroup (in red color).

**Figure 3:**
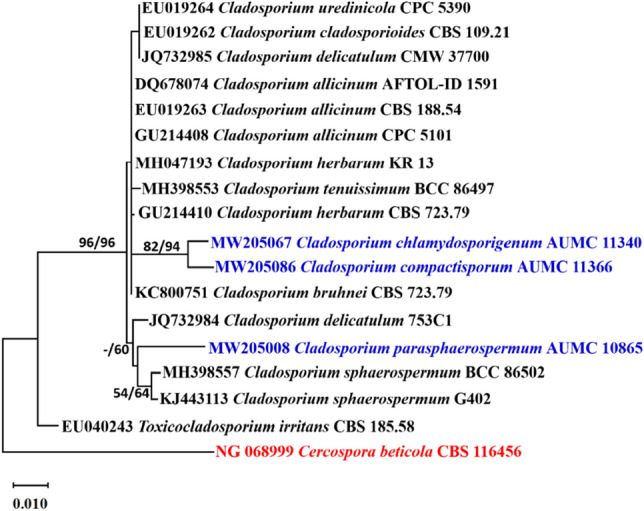
Maximum likelihood phylogenetic tree generated from ML/MP combination analysis based on alignment of LSU sequences of *C. parasphaerospermum* AUMC 10865, *C. chlamydosporigenum* AUMC 11340 and *C. compactisporum* AUMC 11366 with the most similar sequences belonging to *Cladosporium* in GenBank database. Sequences of species in this study are in blue color. Bootstrap support values (1000 replications) for ML/MP combination equal to or greater than 50% are indicated at the respective nodes. The tree was rooted to sequence of *Cercospora beticola* CBS 116456 as outgroup (in red color).

Descriptive statistical parameters of phylogenetic analyses and calculated tree scores for each analyzed sequence locus are summarized in Table 1. The constructed phylogenetic trees for ITS, ACT and LSU are shown in Figs. 1, 2, 3, respectively.

**Table 1:** Statistical parameters representing phylogenetic studies performed on three distinct loci’s sequence alignments.

Parameter	ITS	ACT	LSU
Number of sequences included	32	24	18
Number of alignment positions	566	226	1373
Number of parsimony informative characters (PIC)	29	59	20
Length of tree/number of steps	135	188	83
Consistency index (CI)	0.733333	0.70518	0.777778
Retention Index (RI)	0.953488	0.894977	0.793103
Rescaled consistency index (RC)	0.699225	0.631074	0.616858
Number of equally parsimonious trees retained	8	1	7
Maximum log likelihood	− 1310.26	− 946.35	− 2060.50

## Taxonomy

### *Cladosporium parasphaerospermum* sp. nov

Moharram AM, Zohri AA, Hesham A, Maher MA and Al-Bedak OA.

### MycoBank

MB 844532.

### Etymology

Name refers to globose to subglobose conidia near to that of *C. sphaerospermum*.

### Holotype

Egypt, Beni Suef, Air, Maher MA, AUMC 10865. Ex-type culture: EMCCN: 2062.

### Macroscopic and microscopic characteristics

**Figure 4:**
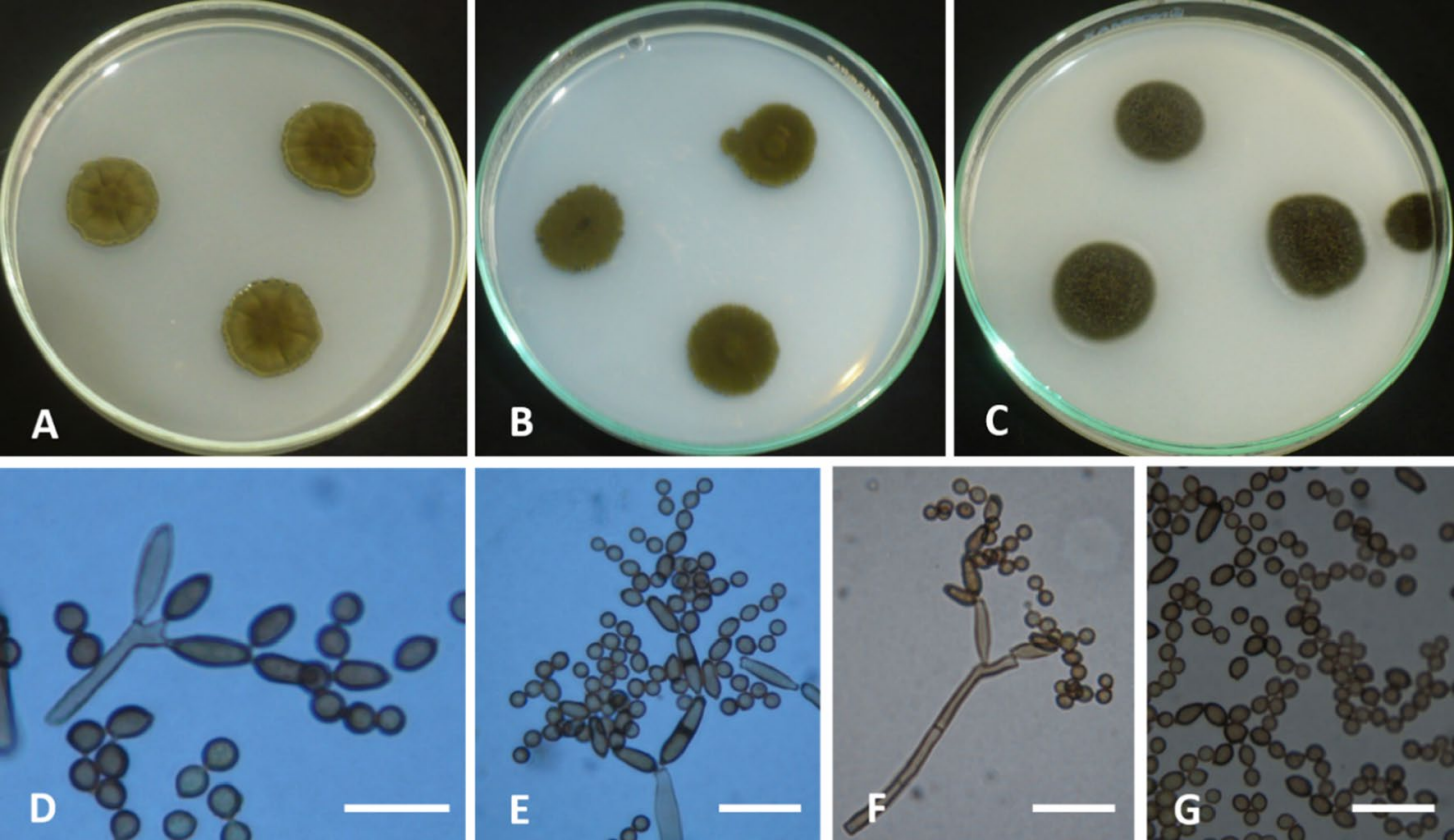
*Cladosporium parasphaerospermum* (AUMC 10865). (**A–C**) 7-day-old colonies on PDA, SNA and OA at 25 °C. (**D–F**) Macronematous conidiophores and conidial chains. (**G**) Globose to subglobose conidia. Scale bar = 20 µm (**D**), 10 µm (**E–G**).

Colonies on PDA reaching 18–20 mm diameter after 7 days at 28 °C, raised at the center, radially sulcate, radially furrowed under the colony, olive, olive green at the center (2F6). Margin curled, paler than the colony center (3F8). Sporulation profuse. Exudates absent. Colonies on SNA reaching 17–19 mm in diameter after 7 days at 28 °C, flat, slightly raised at the center, circular, olive, olive green (3F4-6). Margin entire. Sporulation abundant. Exudates absent. On OA colonies attaining 16–19 mm in diameter after 7 days at 28 °C, circular, flat, somewhat lanuginose, dark olive, dark olive green (1F4-6). Margin entire. Sporulation abundant. Exudates absent. Mycelium abundantly formed, branched, 3–5 µm wide, septate, pale brown to brown, smooth. Conidiophores macronematous, micronematous, abundantly formed, arising terminally or laterally, more or less straight to flexuous, cylindrical, pale brown to brown, smooth, septate, commonly (− 35) 75–100 × 3–5 µm (av. 87.5 × 4) µm (n = 50), not constricted at septa. Ramoconidia integrated, terminal, intercalary, cylindrical, smooth, thick-walled, 0–1 septate, 8–18 × 3–5 µm (av. 13 × 4) µm (n = 50), with 1–3 loci per cell. Loci usually confined to small lateral shoulders, protuberant, conspicuous, short cylindrical, 1–2 µm wide, up to 1–2 µm high. Conidia brown to dark brown, smooth, thick-walled, globose, subglobose, lemon-shaped, 0–septate, 4–6 × 3–5 µm (av. 5 × 4) µm (n = 50). Chlamydospores not formed (Fig. 4).

### *Cladosporium chlamydosporigenum* sp. nov

Moharram AM, Zohri AA, Hesham A, Maher MA and Al-Bedak OA.

### MycoBank

MB 844533.

### Etymology

Refers to the formation of chlamydospores in culture.

### Holotype

Egypt, Sohag, Grapevine fruits, Maher MA, AUMC 11340. Ex-type culture: EMCCN: 2332.

### Macroscopic and microscopic characteristics

**Figure 5:**
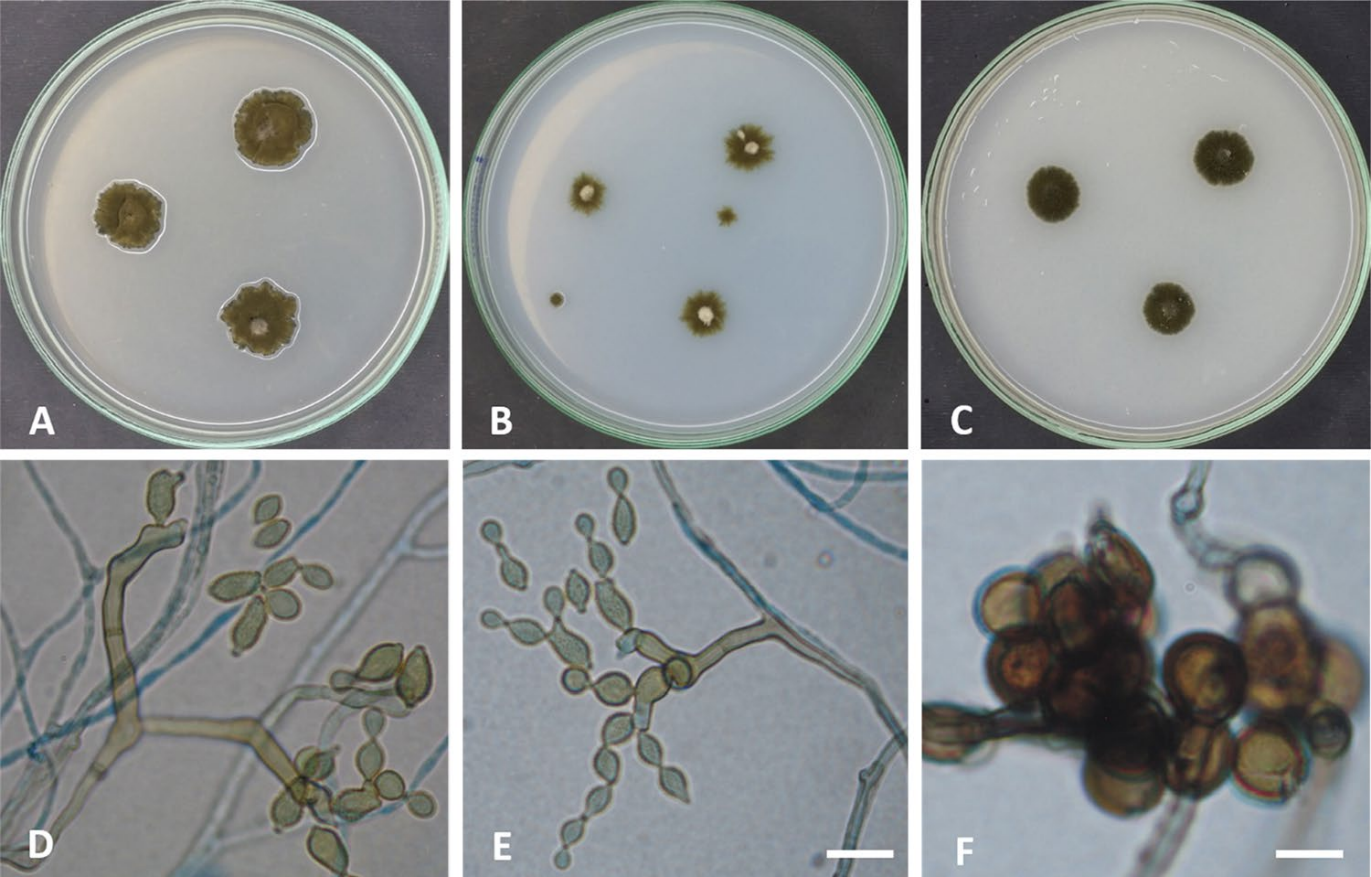
*Cladosporium chlamydosporigenum* (AUMC 11340). (**A–C**) 7-day-old colonies on PDA, SNA and OA at 25 °C. Mycelium with abundant chlamydospores. (**D–E**) Macronematous conidiophores and conidial chains. (**F**) Aggregated chlamydospores. Scale bar = 20 µm.

Colonies on PDA reaching 15–17 mm diameter after 7 days at 28 °C, raised at the center, wrinkled, irregular, olive, olive green (3F6-7). Margin undulate, narrow, paler than the center (3E5-6). Sporulation abundant. Exudates absent. On SNA colonies attaining 9–11 mm in diameter after 7 days at 28 °C, flat, filamentous, olive, olive green (3E2). Margin filiform, narrow, (3E6-7). µm (av. 250 × 5) µm (n = 50), not constricted at septa. Ramoconidia integrated, terminal, intercalary, cylindrical, 11–22 × 6–8 µm (av. 16.5 × 7) µm (n = 50), verruculose to finely roughened, 0–2 septa with 1–3 loci per cell, Loci usually confined to small lateral shoulders, protuberant, conspicuous, short cylindrical, 1–2 µm wide, up to 1 µm high. Conidial chains unbranched or branched, conidia pale brown, straight, subglobose, obovoid to ellipsoid, verruculose to finely roughened, 0–septate, 4–11 × 5–7 µm (av. 7.5 × 6) µm (n = 50). Chlamydospores produced in hyphae, intercalary, aggregated, brown to dark brown, thick-walled, globose, subglobose, 15–25 × µm (Fig. 5).

### *Cladosporium compactisporum* sp. nov

Moharram AM, Zohri AA, Hesham A, Maher MA and Al-Bedak OA.

### MycoBank

MB 844534.

### Etymology

Refers to the compact conidial chains.

### Holotype

Egypt, Qena, Air, Maher MA, AUMC 11366. Ex-type culture: EMCCN: 2358.

### Macroscopic and microscopic characteristics

**Figure 6:**
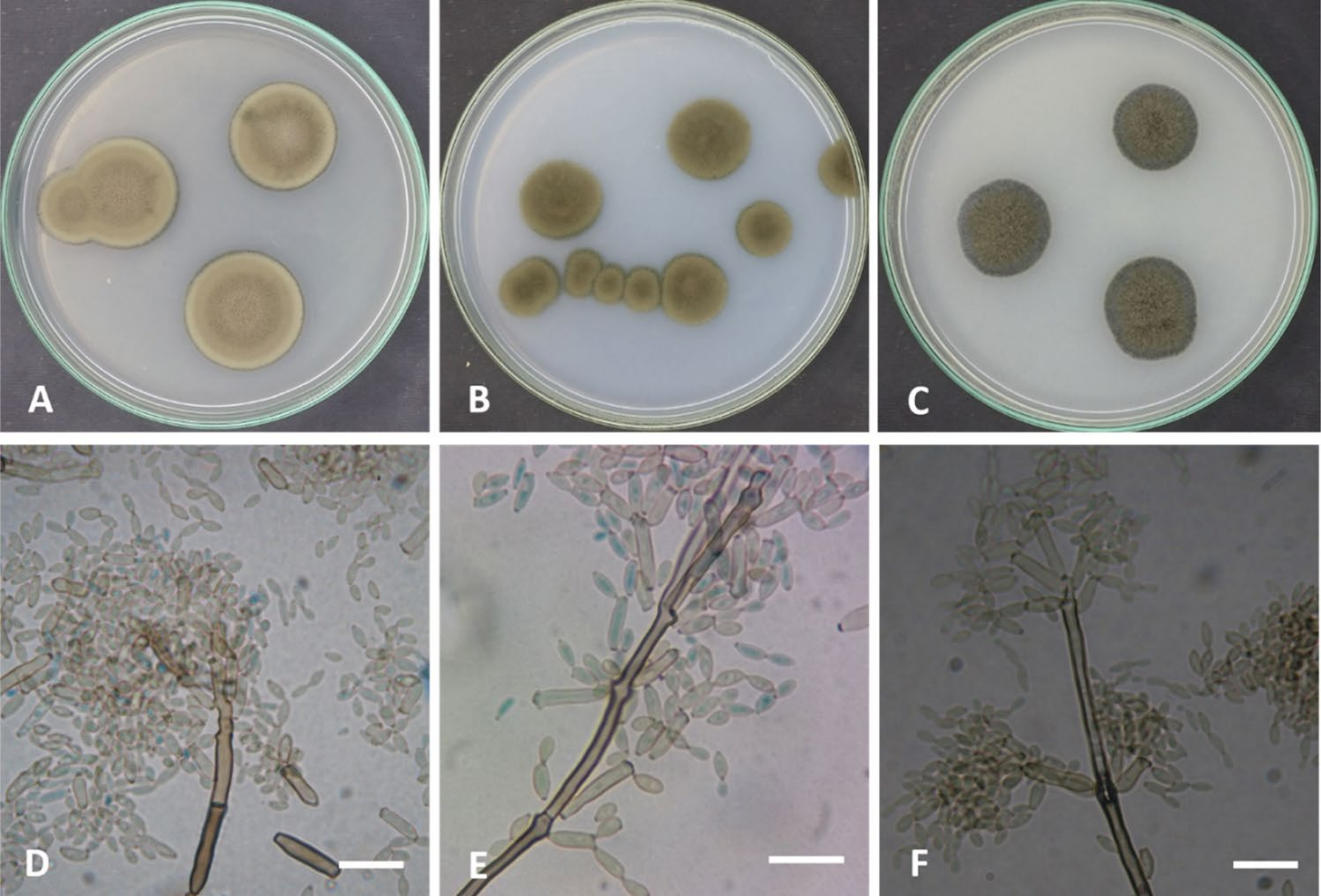
*Cladosporium compactisporum* (AUMC 11366). (**A–C**) 7-day-old colonies on PDA, SNA and OA at 25 °C. (**D–F**) Geniculate and swollen conidiophore bearing compact chains of conidia. Scale bar = 20 µm.

Colonies on PDA attaining 25–28 mm after 7 days at 28 °C, raised, umbonate, circular, olive to olive green (3E3-3F4). Margin entire, narrow, about 3.0 mm in width, paler than the colony center (3E1-3). Sporulation profuse. Exudates absent. Colonies on SNA attaining 17–20 mm diameter after 7 days at 28 °C, raised, umbonate, olive to olive green (3F6-7). Margin entire, about 3.0 mm in width, paler than the colony center (3E2-3). Sporulation abundant. Exudates absent. Colonies on OA attaining 19–23 mm in diameter after 7 days at 28 °C, raised, umbonate, lanuginose, olive grey (2E1-2). Margin undulate, narrow, dark olive grey (2F2). Sporulation abundant. Exudates lacking. Mycelium abundantly formed, branched, 3–5 µm wide, septate, swollen, pale brown to brown, smooth. Conidiophores macronematous and micronematous, abundantly formed, arising terminally or laterally, more or less straight to flexuous, nodulose, geniculate at the upper part, cylindrical, pale brown to brown, smooth, septate, branched, 100–300 µm × 3.0–6.0 µm (av. 200 × 4.5) µm (n = 50). Ramoconidia integrated, terminal, intercalary, cylindrical, 7–22 × 3–4 µm (av. 14.5 × 3.5) µm (n = 50), smooth, 0–1 septa with 1–3 loci per cell. Loci usually confined to small lateral shoulders, protuberant, conspicuous, short cylindrical, 1 µm wide, up to 1–2 µm high. Conidia formed in compact and branched chains, pale brown, subglobose, obovoid to ellipsoid, smooth, 0-septate, 4–6 × 3–4 µm (av. 5 × 3.5) µm (n = 50). Chlamydospores not formed (Fig. 6).

### Optimization of cold-active pectinases production by the three *Cladosporium* strains

#### Effect of pH and temperature on pectinase production

**Figure 7:**
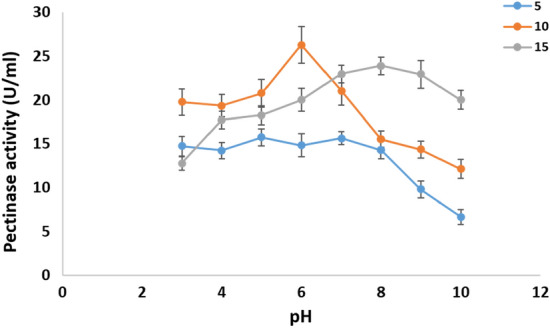
Effect of pH at 5, 10, and 15 °C on the pectinase production by *Cladosporium parasphaerospermum* AUMC 10865.

**Figure 8:**
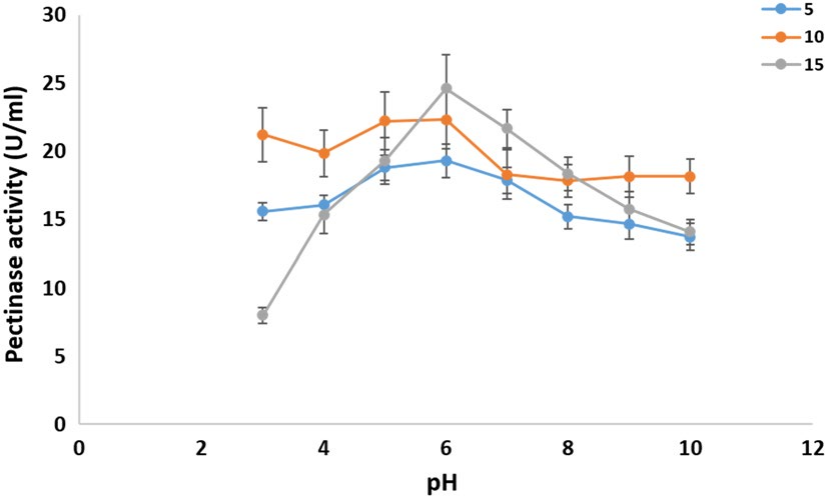
Effect of pH at 5, 10, and 15 °C on the pectinase production by *Cladosporium chlamydosporigenum* AUMC 11340.

**Figure 9:**
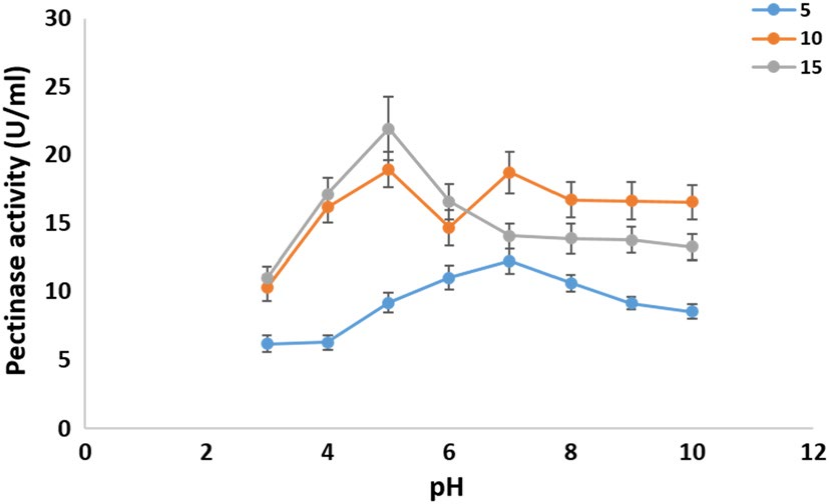
Effect of pH at 5, 10, and 15 °C on the pectinase production by *Cladosporium compactisporum* AUMC 11366.

*Cladosporium parasphaerospermum* AUMC 10865, *Cladosporium chlamydosporigenum* AUMC 11340, and *Cladosporium compactisporum* AUMC 11366 cold-active pectinase production was investigated in this work by altering the pH of the fermentation medium between pH 3.0 and 10.0 each at 5°, 10°, and 15°. *Cladosporium parasphaerospermum* AUMC 10865 produced the most pectinase (26.3 ± 2.1 U/mL) at pH 6.0 and 10 °C (Fig. 7), whereas *C. chlamydosporigenum* AUMC 11340 produced the most (24.63 ± 2.5 U/mL) at pH 6.0 and 15 °C (Fig. 8), and *C. compactisporum* AUMC 11366 (21.93 ± 2.3 U/mL) at pH 5.0 and 15 °C (Fig. 9).

#### Effect of nitrogen source and incubation time

**Figure 10:**
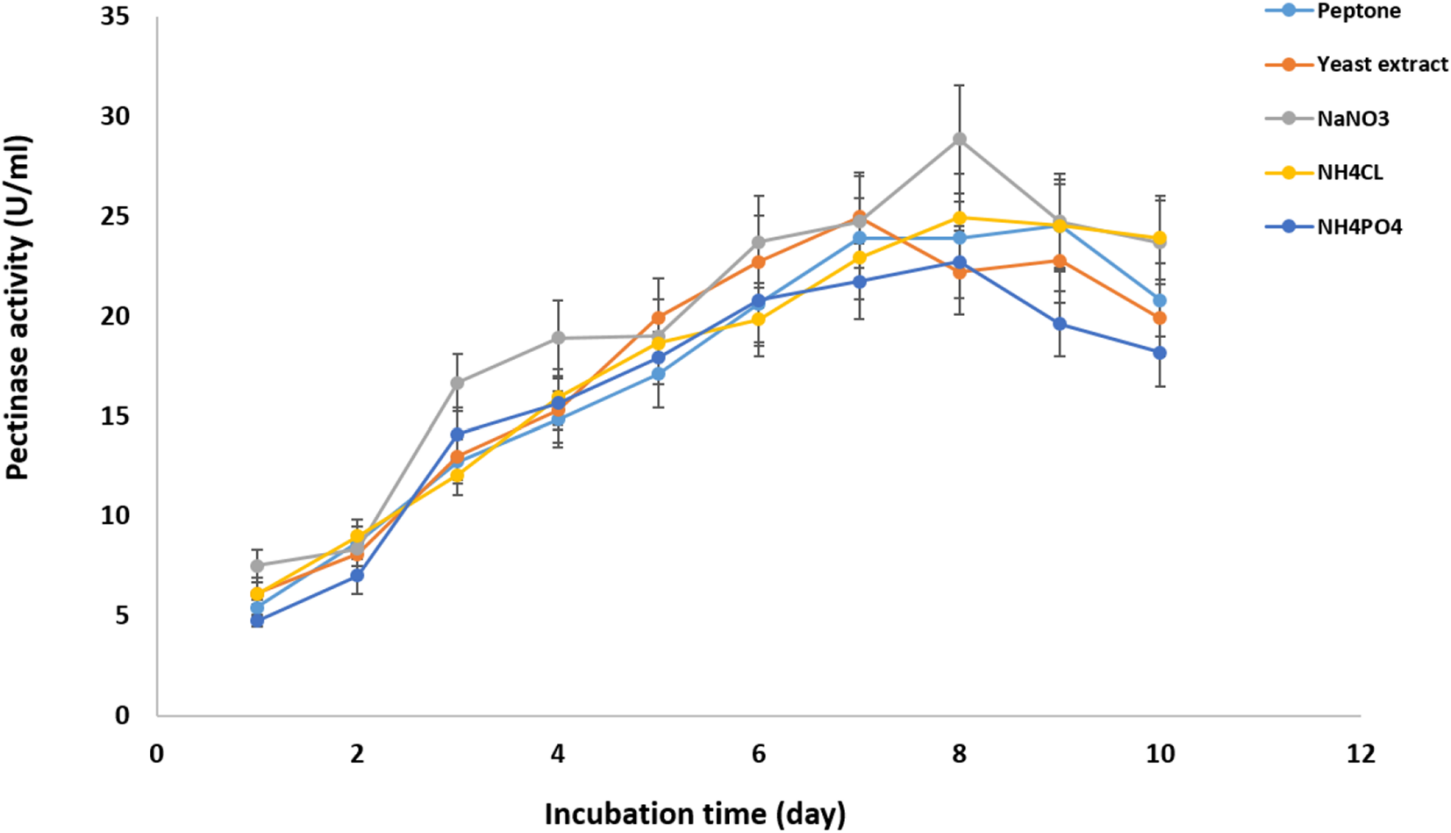
Effect of nitrogen source and incubation time on the pectinase production by *C. parasphaerospermum* AUMC 10865 at pH 6.0 and 10 °C after 8 days.

**Figure 11:**
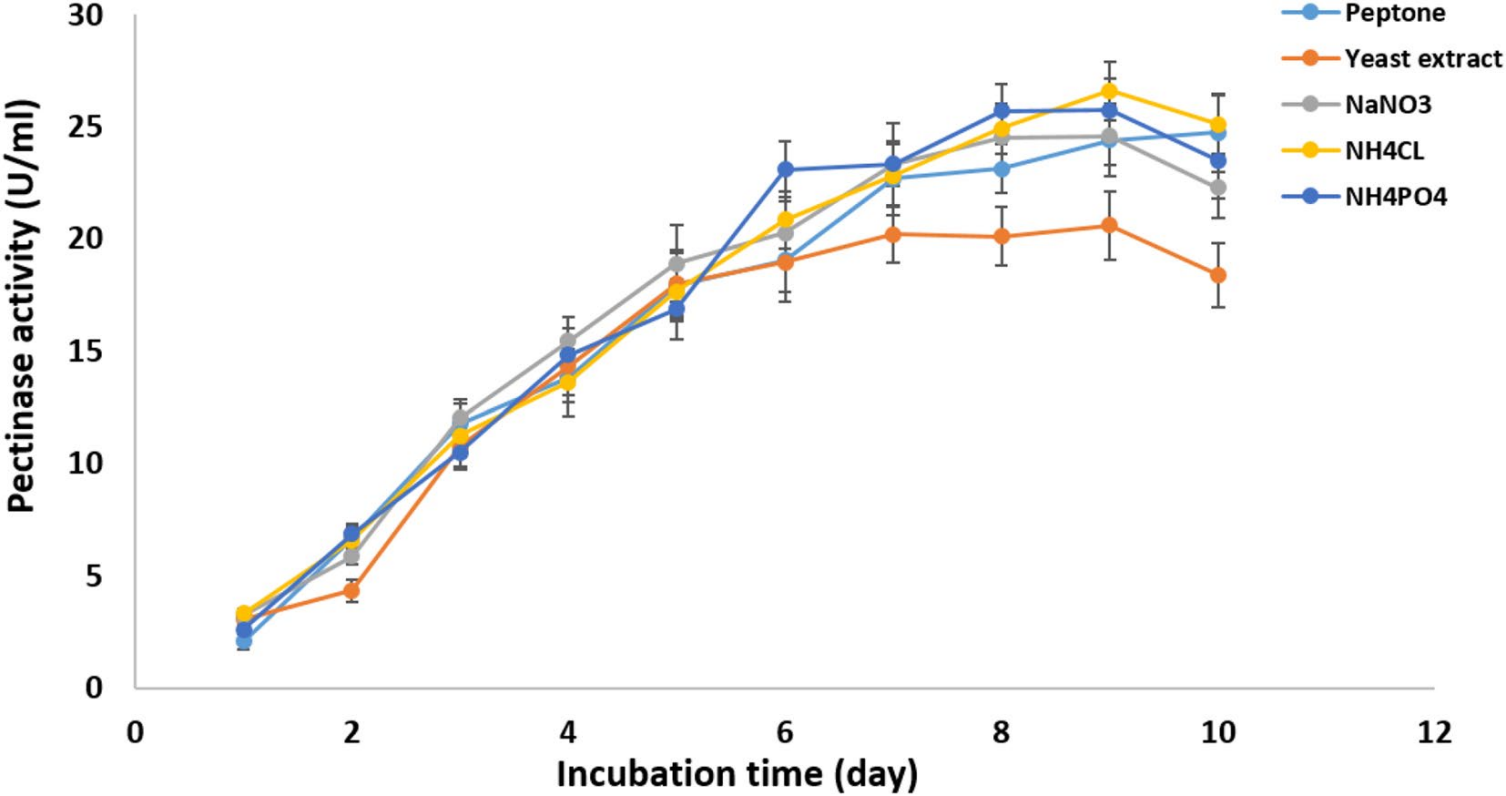
Effect of nitrogen source and incubation time on the pectinase production by *C. chlamydosporigenum* AUMC 11340 at pH 5.0 and 10 °C after 9 days.

**Figure 12:**
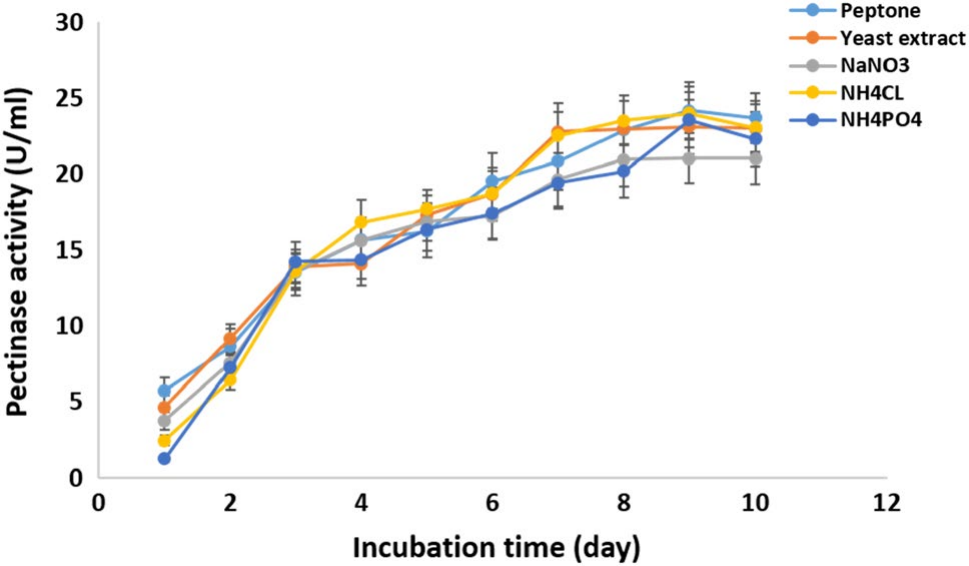
Effect of nitrogen source and incubation time on the pectinase production by *C. compactisporum* AUMC 11366 at pH 7.0 and 5 °C after 9 days.

Pectinase production by *Cladosporium parasphaerospermum* AUMC 10865 was enhanced (28.84 ± 2.7 U/mL) after 8 days incubation using sodium nitrate as a nitrogen source (Fig. 10). While, ammonium chloride was found to be best for pectinase production by *Cladosporium chlamydosporigenum* AUMC 11340 (26.6 ± 1.28 U/mL), and *Cladosporium compactisporum* AUMC 11366 (24.01 ± 1.76 U/mL) after 9 days (Figs. 11, 12).

#### Production of cold-active pectinase by *C. parasphaerospermum* AUMC 10865 in SmF

**Figure 13:**
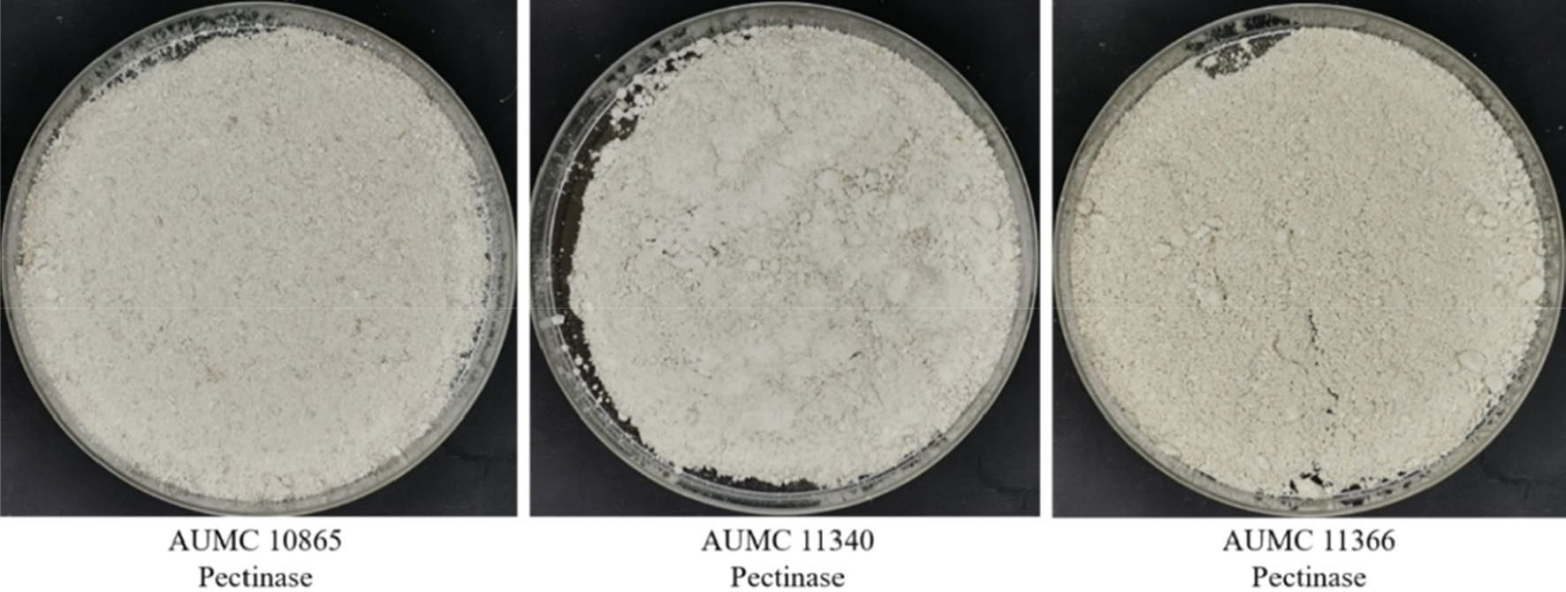
Pectinases powder produced by *Cladosporium* strains in SmF.

In submerged fermentation at the optimum conditions, the three fungi produced pectinases at a rather high output. *Cladosporium parasphaerospermum* generated 5.6 g of pectinase powder per liter of fermentation media, followed by *C. chlamydosporigenum* at 3.65 g and *C. compactisporum* at 2.85 g (Fig. 13). For purification and use, the *C. parasphaerospermum* AUMC 10865 pectinase that was the most cold-active was chosen.

#### Purification of pectinase produced by *Cladosporium parasphaerospermum* AUMC 10865

The *Cladosporium parasphaerospermum* AUMC 10865 pectinase was homogenized using a number of techniques. Initial partial purification of the enzyme involved adding solid ammonium sulphate to the cell-free supernatant. The fraction with a salt saturation of 70% showed pectinase activity. This fraction was dialyzed with citrate buffer (pH 6.0) and freeze-dried before being applied to the anion exchanger (DEAE-Cellulose), which was pre-equilibrated with 50 mM citrate buffer (pH 6.0). The proteins were extracted using a gradient of NaCl (0–1.5 M).

#### Purification profile of pure pectinase

**Figure 14:**
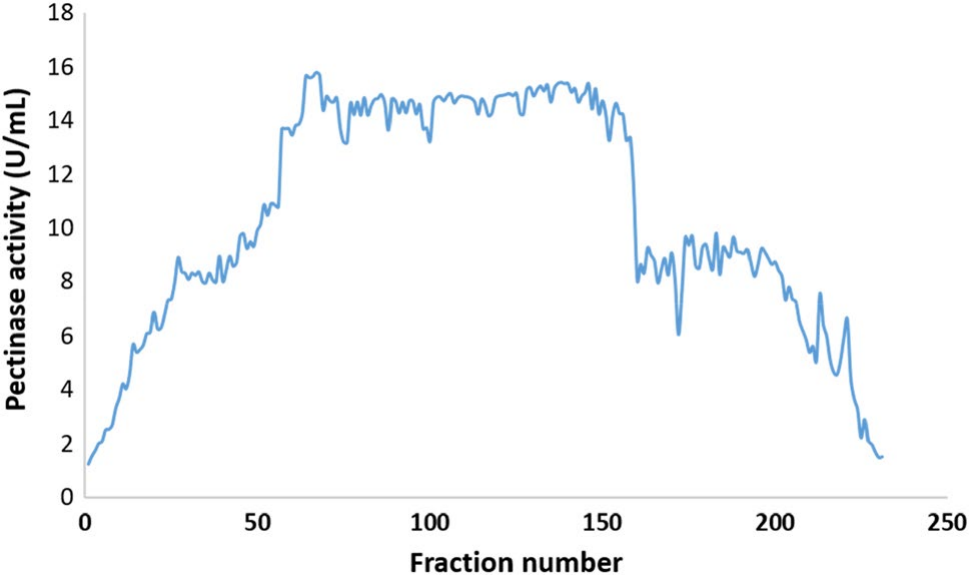
Purification of pectinase by ion exchange chromatography using DEAE-cellulose.

**Figure 15:**
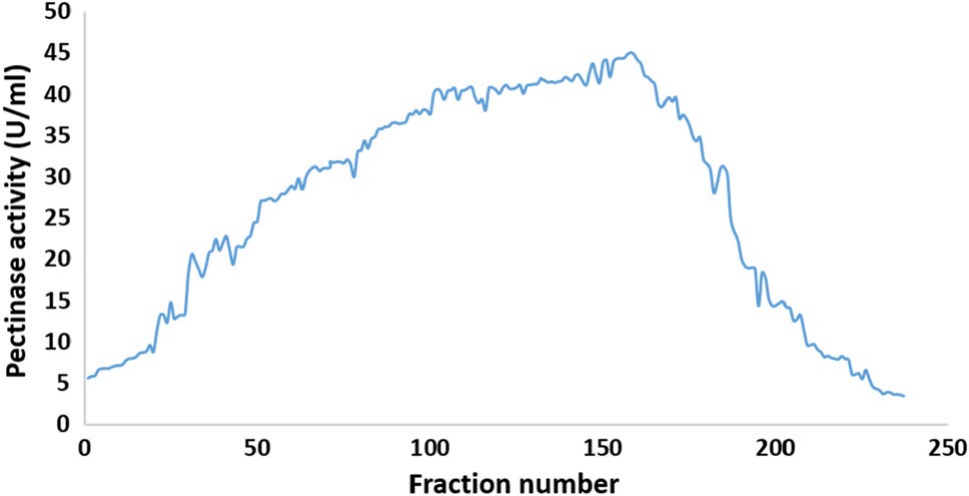
Purification of pectinase by size exclusion chromatography using Sephadex G-100.

Pectinase activity was discovered in fractions 60–150 of a DEAE-Cellulose column (Fig. 14), which were pooled, concentrated, and dialyzed against citrate buffer (pH 6.0). This cycle of purification increased pectinase purity by 214.4-fold, with a specific activity of 1005.55 U/mg protein (Table 2). The fractions with the highest pectinase activity were pooled, condensed with a freeze drier, and loaded onto a Sephadex G-100 column (Fig. 15). The pectinase activity-highest fractions were pooled, concentrated, and dialyzed against citrate buffer (pH 6.0). With a specific activity of 1906 ± 65 U/mg protein, this phase of purification resulted in a 406.4-fold improvement in pectinase purity (Table 2).

**Table 2:** Purification profile of pure pectinase produced by *C. parasphaerospermum* AUMC 10865 at pH 6.0 and 10 °C in SmF.

Purification steps	Fraction volume (mL)	Protein (mg/mL)	Total protein (mg)	Activity (U/mL)	Total activity (U)	Specific activity (U/mg)	Yield (%)	Purification factor (fold)
Fermentation medium	1000	0.1464	146.4	0.687	687	4.692	100	1
Ammonium sulfate	6.5	6.46	42	17.31	112.5	2.68	16.37	0.57
DEAE-Cellulose	15.0	0.018	0.27	18.1	271.5	1005.55	39.52	214.4
Sephadex G-100	10.0	0.003	0.03	5.718	57.18	1906	8.32	406.4

#### Effect of pH and temperature on the pure pectinase activity

**Figure 16:**
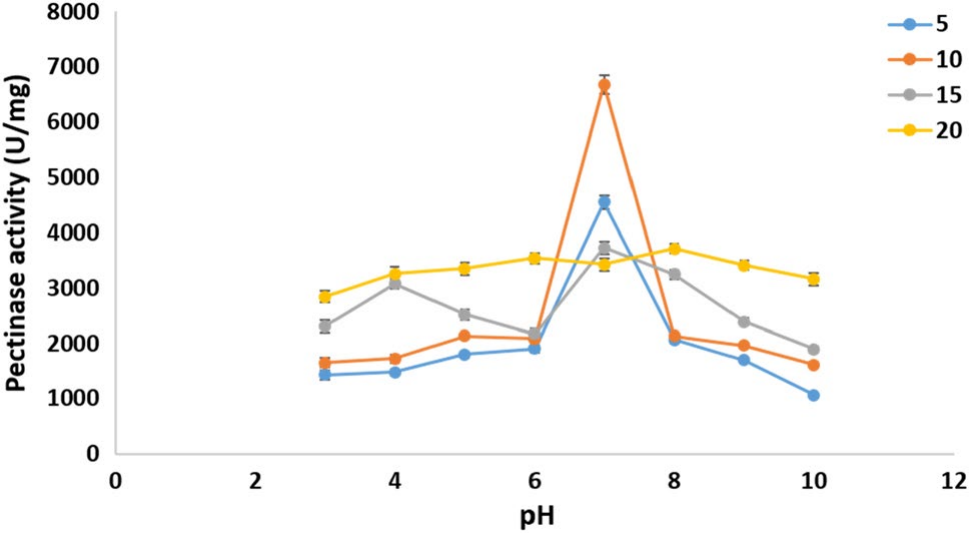
Effect of pH and temperature on activity of the pure pectinase.

The activity of pure pectinase was further tested in the presence of various physical and chemical factors. The purified pectinase has an optimal pH of 7.0. The purified pectinase had the highest activity (4553 ± 124 U/mg) at pH 7.0 and 5 °C, which increased to 6684 ± 173 U/mg at 10 °C (Fig. 16).

#### Effect of some ions and inhibitors on the pure pectinase activity

The purified enzyme was sensitive to all salts tested at a concentration of 5 mmol/mL. Pectinase activity was significantly reduced by 99.21, 96.03, and 94.45% using EDTA, MgCl_2_, and SDS, respectively in the reaction (Table 3).

**Table 3:** Effect of some ions and inhibitors (5 mmol/mL) on pectinase activity produced by *C. parasphaerospermum* (mean ± SD, n = 3).

Ions and inhibitors	Specific activity (U/mg)	Residual activity (%)
**Control**	**6684 ± 173**^**a**^	**100**^**a**^
Na^+^	2387 ± 78^g^	35.7^f^
K^+^	2758.66 ± 69^e^	41.27^e^
Fe^+2^	583.53 ± 43^j^	8.73^i^
Cu^+2^	848.6 ± 66^i^	12.7^h^
Ca^+2^	3130 ± 116^c^	46.83^c^
Mg^+2^	265.2 ± 21^l^	3.97^j^
Zn^+2^	2970.6 ± 117^d^	44.44^d^
Ni^+2^	2652.5 ± 95^f^	39.7^e^
Co^+2^	3554.33 ± 123^b^	53.17^b^
Mn^+2^	1379.33 ± 88^h^	20.63^g^
EDTA	53.0 ± 8^m^	0.79^k^
SDS	371.33 ± 23^k^	5.55^j^

The results are expressed as the activity in the tested inhibitory conditions compared to the pectinase activity in the control without inhibitors (in bold). At the 0.05 level of probability, means in a column with the same letters are not statistically different. Significant values are in bold.

#### Kinetic constants of the pure pectinase

**Figure 17:**
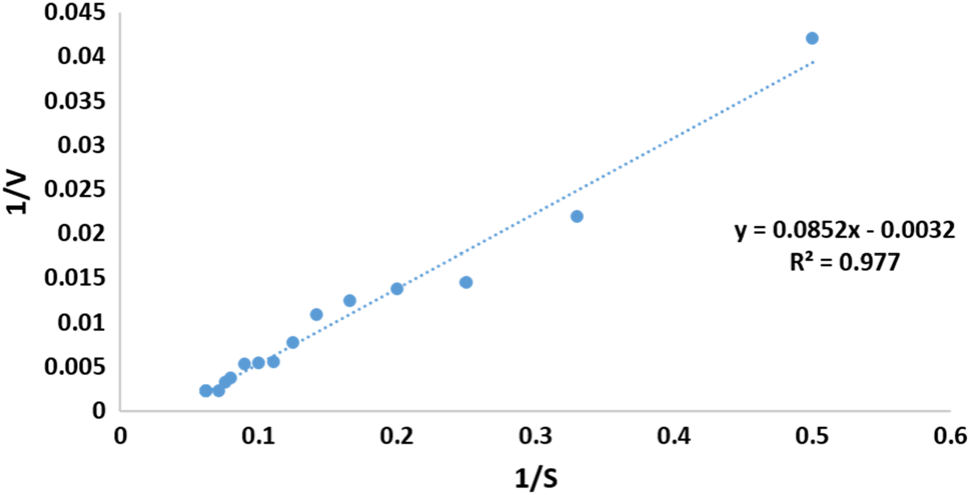
Line-weaver-Burk equation used for K_m_ and V_max_ calculation.

The current findings revealed that Michaelis–Menten constant (K_m_) and the maximum reaction velocity (V_max_) values for the pure pectinase were calculated as 26.625 mg/mL and 312.5 U/min (Fig. 17).

#### Fruit juice production by the pure pectinase

When compared to the control, the enzyme treatment for all of the fruit pulp used resulted in a significant improvement in juice yield, clarity, and colour. It was determined how enzyme treatments affect the extraction of apple, orange, apricot, and peach juices. Enzyme addition increased juice recovery in all fruits. The enzyme treatment of apple, orange, apricot, and peach resulted in a significant increase in juice yield of 16.45, 16.43, 15.93, and 8.73%, respectively. The results also revealed a significant improvement in the clarity and colour of the juice derived from the pulp of the orange fruit, reaching 194.0% and 338.6%, respectively, above the control, while the rest of the fruits employed showed just a modest rise (Table 4).

**Table 4:** Yield, clarity, colour and pH of apple, orange, apricot, and peach fruit juices treated with pure pectinase produced by *Cladosporium parasphaerospermum* AUMC 10865.

Fruits juice	Enzyme concentration	Juice yield (g)	Increase in juice yield (%)	Clarity (OD 660 nm)	Increase in juice clarity (%)	Colour (OD 420 nm)	Increase in juice colour (%)	pH
Apple	Control	13.6^c^	0^e^	0.19^e^	0^e^	0.32^e^	**0**^d^	**5.08**^**b**^
	10U/mL	16.4^a^	17.3^c^	0.21^d^	10.33^b^	0.46^b^	47.26^c^	**4.48**^**e**^
Orange	Control	13.17^d^	0^e^	0.1^h^	0^e^	0.09^g^	**0**^d^	**4.72**^**c**^
	10U/mL	16.43^a^	19.83^b^	0.3^c^	194.0^a^	0.4^b^	338.58^a^	**4.61**^**d**^
Apricot	Control	13.92^c^	0^e^	0.16^g^	0^e^	0.26^f^	**0**^d^	**4.35**^**f**^
	10U/mL	15.93^b^	12.58^d^	0.17^f^	5.52^c^	0.41^d^	58.4^b^	**4.16**^**g**^
Peach	Control	6.64^f^	0^e^	0.31^b^	0^e^	0.75^c^	**0**^d^	**5.76**^**a**^
	10U/mL	8.73^e^	23.93^a^	0.32^a^	3.6^d^	0.75^a^	0^d^	**4.61**^**d**^

At the 0.05 level of probability, means in a column with the same letters are not statistically different.

Significant values are in bold.

## Discussion

Identification of novel species is at the heart of biodiversity research, and in recent years biodiversity efforts have been favouring DNA based method to morphology-based ones^49–53^. Understanding of the microbial composition aids awareness of host–microbe interactions and their environmental function, revealing a complex and delicate balance that can be easily upset^54–56^. Due to the complexity of fungal genomes and the lack of verified databases documenting appropriate biodiversity, such metagenomic studies in fungi are still limited. Identification of novel species is crucial in biodiversity research, which has lately adopted DNA-based methodologies. In this work, we introduced three new *Cladosporium* species as *Cladosporium parasphaerospermum*, *Cladosporium chlamydosporigenum*, and *Cladosporium compactisporum* based on the morphological characteristics as well as phylogenetic analyses of ITS, ACT, and LSU loci.

*Cladosporium parasphaerospermum*, *C. chlamydosporigenum*, and *C. compactisporum* species clades were supported by ITS rDNA and partial actin gene analyses, with *C. parasphaerospermum* and *C. compactisporum* separated in the ACT tree each by a single long branch, and *C. chlamydosporigenum* separated by a single short branch. Because the three strains occupied discrete lineages, the LSU tree validated their uniqueness. *C. parasphaerospermum* can be recognized from *C. halotolerans*^57^, and *C. parahalotolerans*^57^ by its smaller ramoconidia (8–18 µm), which measure 15–37 and 24–37 µm, in both species, respectively. *C. chlamydosporigenum* is distinguished from other *Cladosporium* species in ITS clade by smaller conidia (4–11 µm) and ramoconidia (11–22 µm), as well as the formation of chlamydospores and the absence of head-like swellings with additional intercalary swellings. *C. compactisporum* was discovered in ITS tree as part of a moderately supported clade alongside *C. cladosporioides* and *C. tenuissimum*, and in the ACT tree as part of the *C. salinae* clade on a lengthy distinct branch. It produces smaller ramoconidia (7–22 µm) with 1–3 loci than *C. cladosporioides* (15–50 µm), which has up to 4 loci packed at the tip. *C. compactisporum* differs from *C. tenuissimum*^57^ in that it has geniculate and nodulose conidiophores with compact conidial chains, whereas most *C. tenuissimum* conidiophores are neither geniculate nor nodulose. On Oat agar, *C. tenuissimum* has a longer conidiophore (up to 900 µm) than *C. compactisporum*. Conidiophores of 100–300 × 3–6 µm in length and ramoconidia of 7–22 µm in length distinguish *C. compactisporum* from *C. salinae*^58^, which has weakly differentiated conidiophores (25–50 × 2.5–3 µm) and smaller ramoconidia (9.5–13.5 × 2.5–3.5 µm).

The current research aimed to isolate cold-active pectinases from three novel *Cladosporium* species that could function at low temperatures. The three strains produced a considerable amount of cold-active pectinases, which were active at temperatures as low as 5 and 10 °C. This is the first report of cold-active pectinase production from psychrotolerant *Cladosporium* species that we are aware of.

Pectinase has lengthy been used in commercial food processing to degrade pectin and aid in various processing steps such as liquefaction, clarification, and juice extraction^59^. Pectinases are among the most widely used enzymes, accounting for 40% of all food enzymes^44,60^. It has been demonstrated that certain *Cladosporium* species generate active pectinases^9,25,26,61–63^. However, the synthesis, optimization, purification, and application of a cold-active pectinase in this study is groundbreaking. Due to minor changes in methodology, it is difficult to compare the values of enzyme activity reported by different researches. As a result, comparisons should be made with care.

Because of their biodegradability, non-toxicity, high selectivity, and high yields, microbial enzymes are superior to chemical synthesis^64^. The global enzyme market was valued at $9.9 billion in 2019 and is expected to grow at a 7.1% annual rate from 2020 to 2027^65^. The majority of commercial enzymes, including pectinase, are now mesophilic or thermophilic. In the food sector, and particularly in the fruit processing sector, there has been an increasing desire to replace high-temperature procedures with low-temperature processes. Specific economic and environmental benefits, such as energy savings, retention of biologically inert and aromatic fragrance components, contamination mitigation, and eradication of any residual enzyme activity, which cause deactivation of enzyme when temperature is raised, are driving this shift in trend^34,59,65–67^.

Pectinases released by microorganisms account for approximately 25% of global food enzyme sales. The vast majority of which is derived from filamentous fungi, specifically *Aspergillus niger*^68,69^. It is uncommon for filamentous fungi to produce pectinase activity below 40 °C. This is true even for filamentous fungi that are psychrophilic or psychrotolerant. *Sclerotinia borealis*, a pathogenic fungus prevalent in extremely cold locations that does not grow over 20 °C, generates pectinases with optimal activity at 40 °C^70^. *Mucor flavus* is another example of a psychrotolerant fungus that generates pectinases with optimum activity at 45 °C^71^.

To the best of our knowledge, there is just one case of a filamentous fungus generating pectinases with optimal activity below 40 °C in the literature. *Botrytis cinerea*, a phytopathogenic fungus, generates pectinases with optimum activity between 34 and 37 °C^72^. In this investigation, *Cladosporium parasphaerospermum* produced high quantity of pectinase with the maximum activity at pH 7.0 and 10 °C. Thus, this is the first study to purify and exploit cold-active pectinase from *Cladosporium* species, which might be great candidates for cold-active enzyme synthesis. Industrial pectinases generated from fungi are a blend of pectinolytic enzymes and other proteins. Other commercial processes, such as fruit juice clarity, require only one kind of pectinase activity. As a result, other sources of pectinase must be investigated. The catalytic properties and stability of an enzyme in diverse physio-chemical conditions are important for commercialization.

Pectinase activity in a pure preparation of *Cladosporium parasphaerospermum* was described and evaluated in this work for its potential application in fruit juice clearing. The use of *Cladosporium parasphaerospermum* pure pectinase improved juice recovery in all fruits used. The enzyme treatment of apple, orange, apricot, and peach resulted in a considerable increase in juice output. The results also demonstrated a considerable improvement in the purity and colour of the juice obtained from orange fruit.

## Conclusion

In the current study, three novel *Cladosporium* species were introduced and described as *Cladosporium parasphaerospermum*, *C. chlamydosporigenum*, and *C. compactisporum*. The three novel species appeared to produce cold-active pectinases that had high activity at pH 6.0 and 10 °C, pH 6.0 and 15 °C, and pH 5.0 and 15 °C, respectively, of which *C. parasphaerospermum* pectinase was the most active. The enzyme was purified by 214.4-fold and 406.4-fold by DEAE-Cellulose and Sephadex G 100, respectively. The highest activity of the pure pectinase was gained at pH 7.0 and 10 °C. K_m_ and V_max_ were calculated to be 26.625 mg/mL and 312.5 U/min, respectively. The use of pure pectinase boosted the yield of apple, orange, apricot, and peach juice and improved the clarity and colour of orange juice. We can now add cold-active pectinase production to the long list of *Cladosporium* species that have been identified. We also report three new species that can be used in biotechnological solutions as active microbial pectinase producers. Although further research is needed, these distinct species might be used to decompose difficult and resistant pectinacious wastes as well as clear fruit juices.

## Materials and methods

### Isolation and maintenance of *Cladosporium* strains

Three *Cladosporium* isolates involved in the current study, of which two were isolated from air of Beni Suef and Qena cities and one from fruits of grapevine cultivated in Sohag city, Egypt. Settle plate method^73^ was employed for isolation of *Cladosporium* from air and direct plating technique^74^ for isolation from grapevine fruits. Czapek’s Dox agar was used as an isolation medium. The isolation medium contained (g/L): Sucrose, 30; Na_2_NO_3_, 2; K_2_HPO_4_, 1; KCl, 0.5; MgSO_4_.7H_2_O, 0.5; FeSO_4_, 0.01; ZnSO_4_, 0.01; CuSO_4_, 0.005; Rose Bengal, 0.05; chloramphenicol, 0.25; agar, 15 and the final pH 7.3. The newly discovered strains were preserved as frozen and lyophilized cultures and added to the culture collections of the Assiut University Mycological Centre (AUMC) and the Egyptian Microbial Culture Collection Network (EMCCN) as AUMC 10865 = EMCCN 2062 (Air, Beni Suef, Egypt), AUMC 11340 = EMCCN 2332 (Grapevine fruits, Sohag, Egypt), and AUMC 11366 = EMCCN 2358 (Air, Qena, Egypt). The new species were catalogued in the MycoBank with accession numbers MB844532, MB844533, and MB844534, respectively, along with their descriptions. Nexus file of the sequence alignments for all data sets were uploaded to Tree BASE http://purl.org/phylo/treebase/phylows/study/TB2:S30171?x-access-code=a18696d1afcf659925a63a31c3ebd045&format=html (Study no. 30171).

### Morphological studies of the *Cladosporium* strains

For growth rate determination and phenetic description of colonies, strains were point inoculated on potato dextrose agar (PDA), synthetic nutrient agar (SNA) and oat meal agar (OA)^75,76^, and incubated at 25 °C for 14 days in darkness. Surface colours were rated using the colour charts^77^.

### Molecular identification of the *Cladosporium* strains

#### DNA extraction, PCR and sequencing of ITS, ACT and LSU

DNA isolation of *Cladosporium* isolates AUMC 10865, AUMC 11340 and AUMC 11366 was performed following CTAB method^78^. The universal primers ITS1 and ITS4^79^ were used for amplification of the internal transcribed spacer (ITS) region, ACT783R and ACT512F for amplification of ACT gene^80^, and LROR and LR7^81^ for amplification of the large subunit (LSU). PCR was done following Al-Bedak and Moubasher^82^.

#### Alignments and phylogenetic analyses

Sequences of *Cladosporium* species (ITS, ACT, LSU) in this study were compared to the type and ex-type species in GenBank. MAFFT (version 6.861b) with the default options^83^ was used for alignment of the three sequence sets (ITS, ACT, LSU) in this study. *Cercospora beticola* CBS 116456 was used as outgroup. Alignment gaps and parsimony uninformative characters were optimized by BMGE^84^. Maximum-likelihood (ML) and Maximum parsimony (MP) phylogenetic analyses were performed using PhyML 3.0^85^. The robustness of the most parsimonious trees was evaluated by 1000 replications^86^. The best optimal model of nucleotide substitution for the ML analyses was determined using Akaike Information Criterion (AIC) as implemented in Modeltest 3.7^87^. The phylogenetic tree was drawn and visualized using MEGA X 10.2.6^88,89^. The resulting tree was edited using Microsoft Power Point (2016) and saved as TIF format^9^.

#### Optimization of cold-active pectinase production by the *Cladosporium* strains

In a previous study, the three *Cladosporium* strains (AUMC 10865, AUMC 11340 and AUMC 11366) were found to be capable of producing cold-active pectinases in SmF at 10 °C^27^. For maximization of pectinase production, pH, temperature, nitrogen source and fermentation time influencing pectinase production were optimized by varying parameters using two factors at a time (TFAT) for the three strains. The experiments were conducted in 250 mL Erlenmeyer flasks each with 50 mL fermentation medium (sucrose-free Czapek’s broth) supplemented with 1% citrus pectin as a sole carbon source. The flasks were inoculated separately with spore suspension (1%; v/v) obtained from 7-day-old of *Cladosporium* strains, and incubated under different operating conditions such as pH (3.0, 4.0, 5.0, 6.0, 7.0, 8.0, 9.0, and 10.0), each at 5, 10, and 15 °C, and nitrogen source (peptone, yeast extract, sodium nitrate, ammonium sulfate, and ammonium chloride; each at 0.2%), at 1–10 days of incubation. Three replications of the experiment were performed.

#### Pectinase assay

The colorimetric approach was used to measure pectinase activity^90^. Under static circumstances, 0.5 mL of adequately diluted cell-free supernatant was incubated with 0.5 mL of 1.0% citrus pectin (prepared in 50 mM Na-citrate buffer, pH 6.0) for 20 min at 10 °C. The mixture was boiled for 15 min after 2.0 mL of 3, 5-Dinitrosalicylic acid (DNS) was added. The colour created was evaluated at 540 nm for absorption. The quantity of enzyme that catalyses the synthesis of 1 µmol of galacturonic acid per minute at the standard assay conditions was defined as one unit of pectinase.

#### Production of cold-active pectinase by *Cladosporium parasphaerospermum* in SmF

For pectinases production by *Cladosporium parasphaerospermum* AUMC 10865, the fungus was employed in Erlenmeyer flasks (500 mL) in SmF at the optimum conditions using the fermentation medium. *Cladosporium* species was inoculated with 1.5 × 10^8^ spore/mL spore suspensions obtained from 7-day-old cultures. The incubation period lasted at 10 °C and 150 rpm.

### Purification of the cold-active pectinase

#### Ammonium sulfate precipitation and dialysis

Following the incubation time, cell-free supernatant was recovered by centrifuging at 10,000 rpm for 10 min. At 4 °C, total protein was isolated using 70% saturated solution of ammonium sulphate. A freeze dryer (VirTis, model #6KBTES-55, NY, USA) was used to separate and lyophilize the precipitated protein. Lyophilized protein was dissolved in citrate buffer (pH 6.0) and dialyzed twice for 2 h at room temperature (cutoffs: 12–14 KD) against deionized water, eliminating the water each time, before being refrigerated overnight at 4 °C to remove salts and small molecules. The dialyzed protein was then lyophilized, and used in enzyme characterization experiments as partially purified fungal pectinase.

#### Ion exchange chromatography

A glass column (30 × 2.0 cm; 75 cm^3^ bed volume) was filled with DEAE-Cellulose anion exchanger. After equilibrating the column with citrate buffer (50 mM, pH 6.0), a 6.0 mL sample was loaded onto it. With NaCl concentrations of 0, 0.1, 0.25, 0.5, 1.0, and 1.5 M, the enzyme was eluted with citrate buffer. The volume of the fractions was 5.0 mL. The pectinase activity was assessed using the previous approach. The fractions with the highest pectinase activity were mixed, concentrated, and kept for further study.

#### Gel filtration chromatography

In a glass column, Sephadex G 100 was packaged (55 × 2.5 cm; bed capacity 270 cm^3^). The protein was eluted using citrate buffer (50 mM, pH 6.0) after this column was loaded with the concentrated sample (15 mL). Pectinase activity were evaluated using the techniques described previously in fractions of 5.0 mL volume. The pectinase-positive portions were mixed together, concentrated, and kept for future research.

#### Impact of pH, temperature and some ions and inhibitors on the pure pectinase activity

The impact of pH (3.0–10.0) at 5–15 °C on pure pectinase activity was investigated. The reaction mixture contained 100 µL pure enzyme and 900 µL pectin (dissolved in 50 mM buffer solution). After the reaction time (20 min), the reaction was terminated by introducing 2.0 mL of 3,5-dinitrosalicylic acid (DNS)^90^, and the pectinase activity was determined as previously mentioned. The buffers used were citrate buffer (pH 3.0–6.0), phosphate buffer (pH 7.0–8.0), and glycine/NaOH buffer (pH 9.0–10.0). Also, some ions (Na^+^, K^+^, Ca^+2^, Co^+2^, Ni^+2^, Cu^+2^, Fe^+2^, Mg^+2^, Mn^+2^, and Zn^+2^) were evaluated by introducing them at 5 mM/mL concentrations as NaCl, KCl, CaCl_2_, CoCl_2_, NiSO_4_, CuSO_4_, FeSO_4_, MgSO_4_, MnSO_4_, and ZnSO_4_. A 5 mM/mL ethylenediaminetetraacetic acid (EDTA) and sodium dodecyl sulfate (SDS) were also used to evaluate an enzyme inhibitor. Under standard conditions, the activity of the microbial pectinase in the absence of metal ions or EDTA or SDS was evaluated to define 100% activity. Three replications of the experiment were performed.

#### Determination of kinetic constant (K_m_ and V_max_)

K_m_ (Michaelis–Menten constant) and V_max_ (maximum reaction velocity) values of the purified pectinase were determined by measuring enzyme activity at different concentrations of citrus pectin (1–16 mg/mL), using the Line-weaver-Burk equation^91^.

#### Application of the pure pectinase in fruit juice production

Apple, orange, apricot, and peach pulps were examined for juice production, clarity, colour, and pH using *Cladosporium parasphaerospermum* AUMC 10865’s pure pectinase. Each fruit pulp was treated with 10 U/mL pectinase enzyme (v/v), with untreated fruit pulps serving as controls. The processed fruit pulps were then incubated at 10 °C for 60 min. After inactivating the enzyme by boiling for 5 min, samples were recovered by centrifugation at 5000×*g* for 10 min. for clarity measurements. The juice yield was estimated by dividing the juice mass by the fruit mass^92^.

#### Statistical analysis

Data were subjected to analysis of variance (ANOVA: two-factor with replication) followed by the Duncan’s multiple range test^93^.

## Data Availability

The newly discovered strains were preserved as frozen and lyophilized cultures and added to the culture collections of the Assiut University Mycological Centre (AUMC) and the Egyptian Microbial Culture Collection Network (EMCCN) as AUMC 10865 = EMCCN 2062 (Air, Beni Suef, Egypt), AUMC 11340 = EMCCN 2332 (Grapevine fruits, Sohag, Egypt), and AUMC 11366 = EMCCN 2358 (Air, Qena, Egypt). The new species were catalogued in the MycoBank with accession numbers MB844532 (MycoBank Typification; MBT 10007647), MB844533 (MycoBank Typification; MBT 10007648), and MB844534 (MycoBank Typification; MBT 10007649), respectively, along with their descriptions. Nexus file of the sequence alignments for all data sets were uploaded to Tree BASE http://purl.org/phylo/treebase/phylows/study/TB2:S30171?x-access-code=a18696d1afcf659925a63a31c3ebd045&format=html (Study no. 30171). The datasets generated and/or analyzed during the current study are available in the GenBank repository (https://www.ncbi.nlm.nih.gov/genbank) and MycoBank (https://www.mycobank.org/).
