# Author Correction: Production of cold-active pectinases by three novel *Cladosporium* species isolated from Egypt and application of the most active enzyme

**DOI:** 10.1038/s41598-023-42490-7

**Published:** 2023-09-22

**Authors:** Ahmad Mohamed Moharram, Abdel-Naser Ahmed Zohri, Abd El-Latif Hesham, Hossam E. F. Abdel-Raheam, Mohamed Al-Ameen Maher, Osama Abdel-Hafeez Al-Bedak

**Affiliations:** 1 Department of Botany and Microbiology, Faculty of Science, Assiut University, Assiut, 71511 Egypt; 2 Assiut University Mycological Centre, Assiut University, Assiut, 71511 Egypt; 3 Department of Genetics, Faculty of Agriculture, Beni-Suef University, Beni-Suef, Egypt; 4 Department of Food Sciences, Faculty of Agriculture, Beni-Suef University, Beni-Suef, Egypt

Correction to: *Scientific Reports* 10.1038/s41598-022-19807-z, published online 16 September 2022

The original version of this Article contained errors.

In Table 2, the “Yield” and “Purification factor” data for “Ammonium sulfate”, “DEAE-Cellulose” and “Sephadex G-100” were incorrect. The correct and incorrect values appear below.

Incorrect:

**Table:** 

Purification steps	Fraction volume (mL)	Protein (mg/mL)	Total protein (mg)	Activity (U/mL)	Total activity (U)	Specific activity (U/mg)	Yield (%)	Purification factor (fold)
Fermentation medium	1000	0.1464	146.4	0.687	687	4.692	100	1
Ammonium sulfate	6.5	6.46	42	17.31	112.5	2.68	0.57	16.37
DEAE-Cellulose	15.0	0.018	0.27	18.1	271.5	1005.55	46.493	214.4
Sephadex G-100	10.0	0.003	0.03	5.718	57.18	1906	9.79	406.4

Correct:

**Table:** 

Purification steps	Fraction volume (mL)	Protein (mg/mL)	Total protein (mg)	Activity (U/mL)	Total activity (U)	Specific activity (U/mg)	Yield (%)	Purification factor (fold)
Fermentation medium	1000	0.1464	146.4	0.687	687	4.692	100	1
Ammonium sulfate	6.5	6.46	42	17.31	112.5	2.68	16.37	0.57
DEAE-Cellulose	15.0	0.018	0.27	18.1	271.5	1005.55	39.52	214.4
Sephadex G-100	10.0	0.003	0.03	5.718	57.18	1906	8.32	406.4

In addition, the title of Table 4 was incorrect.

“Yield, clarity, colour and pH of apple, orange, apricot, and peach fruit juices treated with crude pectinase produced by *Cladosporium parasphaerospermum* AUMC 10865.”

now reads:

“Yield, clarity, colour and pH of apple, orange, apricot, and peach fruit juices treated with pure pectinase produced by *Cladosporium parasphaerospermum* AUMC 10865.”

Finally, in the Materials and Methods section, under the subheading ‘Isolation and maintenance of *Cladosporium* strains’, as well as in the Data availability section,

“Nexus file of the sequence alignments for all data sets were uploaded to Tree BASE http://purl.org/phylo/treebase/phylows/study/TB2:S23783 (study no. 29214).”

now reads:

“Nexus file of the sequence alignments for all data sets were uploaded to Tree BASE http://purl.org/phylo/treebase/phylows/study/TB2:S30171?x-access-code=a18696d1afcf659925a63a31c3ebd045&format=html (Study no. 30171).”

The original Article has been corrected.

